# Recurrence of Angioimmunoblastic T-cell Lymphoma in a Patient Successfully Treated for Hodgkin’s Lymphoma: A Case Report

**DOI:** 10.7759/cureus.29867

**Published:** 2022-10-03

**Authors:** Syed Alishan Nasir, Simranjit Johal, Andrew White, Bhavna Khandpur, Daniel Boxer

**Affiliations:** 1 Internal Medicine, Norwalk Hospital, Norwalk, USA; 2 Internal Medicine, Ross University School of Medicine, Norwalk, USA; 3 Pathology, Norwalk Hospital, Norwalk, USA; 4 Hematology and Oncology, Norwalk Hospital, Norwalk, USA

**Keywords:** t cell neoplasm, t-cell lymphoma, relapse lymphoma, non-hodgkin-s lymphoma, adult hodgkin lymphoma survivor, hodgkin lymphona

## Abstract

Hodgkin’s lymphoma (HL) is a common and potentially curable malignancy that has an overall good prognosis when timely treatment with chemoradiation is delivered. Recurrence of malignancy is one complication seen in patients successfully treated for HL. In most cases, the recurring malignancy can be a solid tumor or leukemia. While recurrence of a non-HL (NHL) has been reported, this is relatively uncommon. Angioimmunoblastic T-cell lymphoma (AITL) is a rare nodal appearing, peripheral T-cell lymphoma and represents 2% of all NHLs. Its clinical features include generalized lymphadenopathy, varying constitutional symptoms, and autoimmune-related hematologic findings, such as hemolytic anemia and or thrombocytopenia. Diagnosis is made based on histological and immunohistochemical (IHC) findings, which show evidence of T-cells, follicular T-cell markers, and characteristic genomic features including mutations of T-cell receptor or T-cell receptor signaling genes. It is a characteristically aggressive cancer with a poor prognosis if untreated and therefore requires prompt diagnosis. While sporadic AITL is rare on its own, data on AITL occurrence in patients previously treated for HL is lacking. We present a peculiar case of an 80-year-old patient who was diagnosed and treated for stage IV Hodgkin’s disease only to be later diagnosed with AITL.

## Introduction

Angioimmunoblastic T-cell lymphoma (AITL) is a predominantly nodal appearing T-cell neoplasm. Initially regarded as a premalignant, non-neoplastic lymphoid process with a tendency to develop into a lymphoma, called angioimmunoblastic lymphadenopathy with dysproteinemia (AILD), it became established as a neoplastic disease in the 1980s after the identification of clinical cytogenetic abnormalities of the clonal T-cell receptor gene rearrangements [1]. Later, it was recognized as angioimmunoblastic T-cell lymphoma (AITL) in the revised European-American classification of lymphoid neoplasms (REAL) and World Health Organization (WHO) classifications [1]. It is a rare disease and accounts for approximately 15%-30% of peripheral nodal T-cell lymphomas (PTCL) globally and represents 2% of all non-Hodgkin’s lymphomas (NHLs) [1,2]. No consistent risk factors or etiological agents have been identified in AITL [1]; however, there is some association with Epstein-Barr virus (EBV) and several reports have shown that high EBV viremia upon presentation was associated with a worse response, disease progression, or evolution into aggressive B-cell lymphoma [2]. The diagnosis of AITL is accomplished by pathologic analysis, with immunohistochemistry (IHC) being of utmost importance. Presenting symptoms include, and are not limited to, systemic constitutional symptoms such as fevers, night sweats, anorexia, and acute onset lymphadenopathy. Treatment is delivered with the goal of curing and is similar to treatment used for other nodal PTCLs with CHOP (cyclophosphamide, doxorubicin, vincristine, prednisone) being the first line. Despite treatment however both low-risk and high-risk subgroups of patients with AITL have been shown to have a five-year survival of 44% and 24%, respectively [2]. Herein we discuss a rare case of a patient who was diagnosed and treated for stage IV Hodgkin’s disease only to have a recurrence with AITL.

## Case presentation

**Figure 1:**
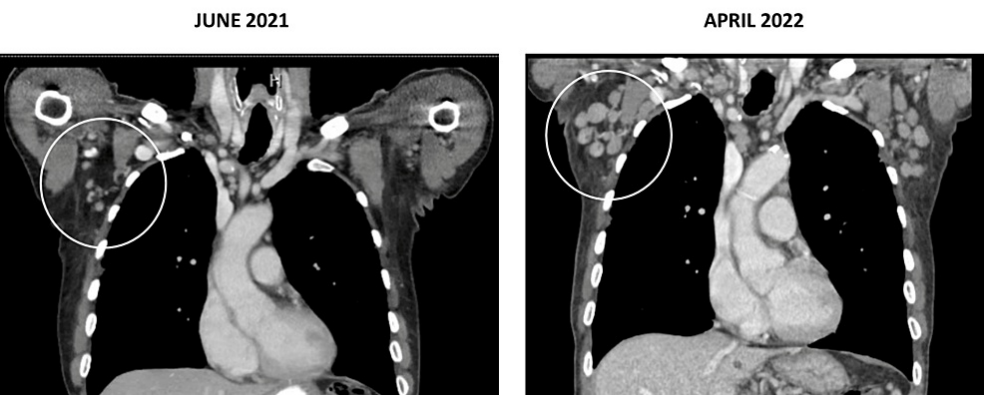
Chest CT scan showing the change in axillary lymphadenopathy from June 2021 to April 2022

**Figure 2:**
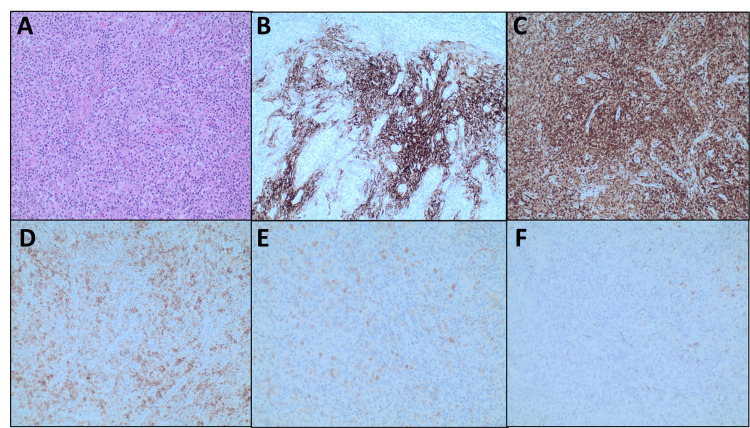
Pathology specimens from lymph node biopsy A: H+E - 10x image showing effacement of architecture, polymorphous inflammatory background, and prominent vascularity, B: CD21 immunostain - 10x image showing dendritic meshwork and neoplastic cells in clusters surrounded by dendritic processes highlighted by CD21, C: CD4 immunostain - 10x image showing neoplastic T helper cell phenotype, D: ICOS immunostain - 10x image showing neoplastic T helper cell phenotype, E: PD1 immunostain - 10x image showing neoplastic T helper cell phenotype, F: CXCL13 immunostain - 10x image showing rare positive cells.

We present the case of an 80-year-old male, with stage IV Hodgkin’s lymphoma (HL), who was initially treated with three cycles of Adriamycin 25mg/m^2^ intravenous (IV) push, Bleomycin 10units/m^2^ IV over 10 minutes, Vinblastine 6mg/m^2^ IV over 10 minutes and Dacarbazine 375mg/m^2^ IV for 10 minutes (ABVD) delivered on day 1 and day 15 per cycle, followed by a transition to brentuximab 1.8mg/kg IV every 21 days due to bleomycin related pulmonary adverse effects and pancytopenia. He completed a total of four cycles with brentuximab after which the treatment was discontinued due to severe polyneuropathy and the patient was being observed off treatment. The patient was off therapy since March 2021; however, in April 2022, presented to the outpatient oncology clinic with complaints of worsening lethargy and decreased appetite. In response to his change in health, a computed tomography (CT) scan of the chest, abdomen, and pelvis was obtained and showed an interval increase in axillary, mediastinal, inguinal, and abdominal lymphadenopathy in comparison to the prior CT scan from 2021 as shown in Figure 1. The initial suspicion was for recurrence of HL and an ultrasound guide core axillary lymph node biopsy was obtained; however, it was noted to be non-diagnostic. The initial plan was to remain off Hodgkin’s directed therapy and follow up in three months’ time; however, four weeks later patient began experiencing night sweats, developed tender axillary lymphadenopathy, and increased fatigue. During this time, he also had a weight loss totaling 8-10 lbs. A total body positron emission tomography (PET) was obtained which showed an increased number of borderline-sized axillary lymph nodes and low-grade activity within the right paratracheal, left hilar, and bilateral inguinal lymph nodes. An excisional biopsy of a right axillary lymph node was then performed, and pathology (Figures 2A-2F) demonstrated effacement of architecture due to paracortical expansion of atypical small lymphoid cells with clear cytoplasm. No Reed-Sternberg cells were identified and immunostains for CD4, and CD8 showed a predominance of CD4-positive T cells. Stains were positive for CD21, PD1, ICOS, and CXCL13. A Ki-67 stain showed a proliferation rate of 30%-40% and T-cell gene rearrangement was noted to be positive. Based on the pathological findings, our patient was diagnosed with AITL and promptly started on therapy with reduced dose CHOP (cyclophosphamide 400mg/m^2^ IV push, doxorubicin 25mg/m^2^ IV push, vincristine 1mg IV infusion over 5-10 minutes, and prednisone 40mg oral). Each cycle was 21 days long and he received the cyclophosphosphomide, doxorubicin, and vincristine on day 1 only in addition to the prednisone from day 1 to day 5. He underwent two complete cycles during which the patient noted an overall improvement in night sweats and lymphadenopathy. Unfortunately, shortly after finishing his second cycle, he was hospitalized for an ischemic stroke with left-sided vision loss. His hospital course was complicated by aspiration pneumonia and septic shock due to which the patient ultimately passed away.

## Discussion

AITL is an uncommon subtype of mature PTCL. Since its first description in 1970, the incidence of AITL has remained unchanged, in that, it only accounts for approximately 1% to 2% of all NHL with variable geographical incidence (16% in the USA, 18% in Asia, and 29% in Europe) [2]. A large population-based study using the Surveillance, Epidemiology, and End Results (SEER) database reported 1207 AITL patients, the median age at diagnosis was 69 years. The incidence was slightly higher in males (51.5%) and advanced-stage (III to IV) disease was reported in 80% of patients [3]. AITL also poses a poor prognosis with one study showing overall survival (OS) probabilities at two, five, and 10 years at 46.8%, 32.9%, and 21.9%, respectively, as well as two-year, five-year, and 10-year disease-specific survival (DSS) rates at 56.1%, 44.0%, and 35.9%, respectively, with no survival improvement for patients over the past two decades [3]. Historically, AITL has been referred to by many different names and while initially thought to be a non-neoplastic immunoreactive/inflammatory process called AILD, it received the designation of a true lymphoid neoplasm by the WHO in 2008 [4]. There is no consistently reported etiology or risk factor that has been linked to the development of AITL. While EBV infections have been associated [2], data around prior malignancies and treatment as a risk factor, has been lacking. In our literature review, we could not find any reports that described prior malignancies and a history of chemoradiation therapy as a risk factor for developing AITL. Furthermore, we also did not find any reports where a recurrence of AITL was documented in patients with prior history of lymphoid malignancies.

AITL patients are typically symptomatic upon presentation. The most common clinical complaint seen in up to 70% of patients is B symptoms (fevers, unintentional weight loss, and night sweats) [1,4]. Bone marrow involvement is observed in up to 70% of the patients and tends to correlate with a higher frequency of B-symptoms, hepatosplenomegaly, laboratory abnormalities, and with the presence of circulating tumor cells [1,2,4]. Generalized peripheral lymphadenopathy is commonly seen. Hepatomegaly is seen in a modest proportion [4]. Extranodal involvement including skin manifestations such as rashes, seen in 20%-50%, urticaria, abscesses, ulcers, and nodular tumors have also been reported [4,5]. Immune activation with elevated sedimentation rate, and positive autoimmune tests such as rheumatoid factor, anti-smooth muscle, monoclonal gammopathy, and warm autoimmune hemolytic anemias have also been reported [4]. Other clinical signs and symptoms, such as arthralgias or arthritis, pleural effusions, ascites and/or edema, neurological manifestations, and gastrointestinal symptoms, are also seen, but less common. Peripheral blood leukocytosis with lymphocytosis is rare; however, the presence of aberrant T-cells may be detected by flow cytometry [1].

In patients with AITL, pathological analysis of lymph nodes shows complete structural effacements with the presence of inflammatory cells, normal CD4+ and CD8+ ratio, and cells positive for CD3, CD4, and CD5 [6]. Immunohistochemical staining shows positivity for CXCL13 which has been reported in 76%-100% of cases, PD1 (also known as CD279) reported to be positive in 62%-100% of cases and inducible T-cell costimulatory (ICOS) reported to be positive in 30%-89% of cases [6,7]. Pathology from our patient’s biopsy revealed positivity for all three markers. A retrospective study of 207 patients with AITL performed by Tokunaga et al. showed age older than 60 years, elevated white blood cell (WBC), elevated IgA levels, the presence of anemia and thrombocytopenia, and extranodal involvement at > 1 site were significant prognostic factors for overall survival, and IgA, anemia, and mediastinal lymphadenopathy were significant prognostic factors for progression-free survival [7]. Among pathological prognostic factors, Ki67 proliferation > 30% and transformed tumor cells > 20% were adverse predictors of overall survival [8]. Despite treatment, AITL is characterized by an aggressive course and abysmal outcome [8]. Most patients die of infectious complications rather than tumor load, suggesting that an underlying immunodeficiency significantly contributes to AITL-associated mortality [9].

Options for treatment are limited and the gold standard therapy is CHOP despite retrospective evidence that shows a lack of efficacy of anthracycline-based therapies. In AITL, CHOP carries a complete response rate of about 53% [10]. Our patient was treated with a reduced dose of CHOP (mini-CHOP). In some cases, Rituximab, an anti-CD20 monoclonal antibody, has also been employed in combination with CHOP. In a phase 2 study in which 25 patients underwent rituximab-CHOP therapy for six cycles, the overall response rate was 80% with 44% complete response. The two-year overall survival rate was 62% [11]. Another monoclonal antibody, alemtuzumab (anti-CD52) was combined with CHOP in several trials. The reported response rates were possibly better than CHOP alone, but patients receiving alemtuzumab developed significant opportunistic infections [2]. Other treatment options have been studied however data supporting their use is not strong. Some examples of treatment options include ACVBP (doxorubicin, cyclophosphamide, vindesine, bleomycin, and prednisone), PEGS (cisplatinum, etoposide, gemcitabine, methylprednisolone), CEOP (cyclophosphamide, etoposide, vincristine, prednisone, and pralatrexate), CHOP plus bevacizumab, belinostat-CHOP therapy, brentuximab vedotin plus CHOP therapy and hypomethylating agents [1-4,6]. Regardless of treatment options, the overall prognosis and long-term survival in these patients remain poor.

A complication frequently encountered after treatment of HL is recurrence and/or development of secondary malignancies. This is in part due to the chemotherapy or radiation therapy that patients receive. Multiple studies have demonstrated an increased risk of secondary malignancies in patients with HL and these are one of the highest causes of mortality in long-term survivors. Secondary malignancies include both solid tumors and hematological malignancies including myelodysplasia, leukemia, and secondary lymphomas. Radiation therapy is associated with an increased risk of malignancy at irradiated sites, such as breast cancer, whereas chemotherapy is associated with secondary leukemia, NHL, and lung cancer [12,13]. In a study by Henry-Amar et al. the 15-year cumulative incidence rate of second cancer was 11.2%. It was 2.2%, 1.8%, and 7.5% for recurrence of acute leukemia, NHL, and solid tumor respectively. Solid tumors make up the major burden of secondary malignancies however data supporting this was mostly derived from older studies when patients received mantle-field radiation therapy and MOPP (mechlorethamine hydrochloride, oncovin, procarbazine hydrochloride, and prednisone) chemotherapy, both of which are not commonly used anymore [12-14].

Notably, the recurrence of NHL is much less frequent than acute leukemia in patients previously treated for and/or cured of HL. In several studies, the incidence of NHL ranges from 1% to 5.9% [13]. Factors associated with an increased risk of secondary NHL include age older than 30, male gender, and clinical stage III HL [14]. The mechanism underlying this onset of NHL from previously treated HL remains unclear but is thought to include cytotoxic effects of therapy, histological conversion of HL, and defective immune surveillance [12]. A retrospective analysis of patients treated within clinical trials of the German Hodgkin’s Lymphoma Study Group from 1991 to 1998 demonstrated that the majority of secondary NHLs were B-cell lymphomas, particularly diffuse large B cell lymphoma, and the prognosis was worse than primary lymphomas. Moreover, the occurrence of secondary T-cell lymphomas was rare and associated with a very poor prognosis with most patients dying within 28 months after diagnosis [15]. In the same study, the outcome of secondary NHL was also heavily influenced by the time of occurrence after HL treatment. Patients that develop NHL within three months after completing their treatment were shown to have a lower overall two-year survival. During our literature review, we found that AITL was not a common recurrence seen in patients treated for HL. Furthermore, we did not come across any reports that documented the onset of AITL in a patient who had been treated for HL, thus making our case a rare and unique presentation.

## Conclusions

AITL is an aggressive T-cell lymphoma associated with high mortality and poor prognosis. Not only is it a rare cause of sporadic NHL, but it is also even rarer to encounter AITL in patients who have already been treated for HL. We therefore present this case to highlight an unusual clinical presentation of AITL and assert that any sign of cancer recurrence in patients with HL should prompt concern for this aggressive and potentially lethal neoplasm and diagnostic investigations must be expedited in order to initiate treatment under the event that patient is found to have AITL.
